# Growth of bacteria in 3-d colonies

**DOI:** 10.1371/journal.pcbi.1005679

**Published:** 2017-07-27

**Authors:** Xinxian Shao, Andrew Mugler, Justin Kim, Ha Jun Jeong, Bruce R. Levin, Ilya Nemenman

**Affiliations:** 1 Department of Physics, Emory University, Atlanta, GA 30322, USA; 2 Department of Physics and Astronomy, Purdue University, West Lafayette, IN 47907, USA; 3 Department of Biology, Emory University, Atlanta, GA 30322, USA; 4 Initiative in Theory and Modeling of Living Systems, Emory University, Atlanta, GA 30322, USA; University of Chicago, UNITED STATES

## Abstract

The dynamics of growth of bacterial populations has been extensively studied for planktonic cells in well-agitated liquid culture, in which all cells have equal access to nutrients. In the real world, bacteria are more likely to live in physically structured habitats as colonies, within which individual cells vary in their access to nutrients. The dynamics of bacterial growth in such conditions is poorly understood, and, unlike that for liquid culture, there is not a standard broadly used mathematical model for bacterial populations growing in colonies in three dimensions (3-d). By extending the classic Monod model of resource-limited population growth to allow for spatial heterogeneity in the bacterial access to nutrients, we develop a 3-d model of colonies, in which bacteria consume diffusing nutrients in their vicinity. By following the changes in density of *E. coli* in liquid and embedded in glucose-limited soft agar, we evaluate the fit of this model to experimental data. The model accounts for the experimentally observed presence of a sub-exponential, diffusion-limited growth regime in colonies, which is absent in liquid cultures. The model predicts and our experiments confirm that, as a consequence of inter-colony competition for the diffusing nutrients and of cell death, there is a non-monotonic relationship between total number of colonies within the habitat and the total number of individual cells in all of these colonies. This combined theoretical-experimental study reveals that, within 3-d colonies, *E. coli* cells are loosely packed, and colonies produce about 2.5 times as many cells as the liquid culture from the same amount of nutrients. We verify that this is because cells in liquid culture are larger than in colonies. Our model provides a baseline description of bacterial growth in 3-d, deviations from which can be used to identify phenotypic heterogeneities and inter-cellular interactions that further contribute to the structure of bacterial communities.

## Author summary

The vast majority of theoretical and experimental studies assume that bacteria exist as planktonic cells in well-mixed liquid cultures, all with equal access to nutrients, wastes, toxins, antibiotics, bacterial viruses, and each other. However, in the real world, bacteria are more often found in physically structured, spatially heterogeneous habitats as colonies and micro-colonies. While one can experimentally explore the population and evolutionary dynamics of bacteria in such physically structured habitats, there is dearth of mathematical models to generate hypotheses for and to interpret results of these experiments. As a step towards the construction of a theory of the population dynamics of bacteria in physically structured habitats, we develop and experientially explore the simplest such model of the dynamics of bacterial growth in 3-d structured colonies.

## Introduction

In 1942, Jacques Monod developed a mathematical model of bacterial growth in a liquid culture, where the bacterial cells and nutrient molecules were homogeneously distributed [1, 2]. A simple ordinary differential equation was accurate enough to account for the exponential growth of bacteria and their ascent into stationary phase following the exhaustion of the limiting resource. The model has proven to be long-lived since most experimental studies of the population dynamics of bacteria are in liquid culture [3, 4]. In contrast, outside the tubes, flasks, and chemostats of laboratory culture, bacteria most commonly live in physically structured habitats as colonies or microcolonies. Such colonies are heterogeneous; at a minimum, cells vary in their access to nutrients depending on their position within the colony and thereby divide at different rates.

The majority of research directed at understanding structured bacterial population growth has been confined to two dimensional (2-d) surfaces [5–10], including studying the interplay of evolution and the physical structure [11, 12], or analyzing effects of mechanical interactions in an expanding colony [13–15]. However, diffusion in two dimensions is different from three, making it easier to form diffusion-limited instabilities [5, 16, 17]. In 3-d, work has been done to understand nutrient shielding of the interior of a colony by the microbes on the surface, treating them as individual agents [18]. In addition, in the context of modeling biofilms, there exist many complex models that account for mechanical stresses, adhesion properties, fluid flows, fluxes of multiple metabolites and waste product, and so on (see Refs. [19–26] for just a few examples). These models typically involve many free parameters, not all of them experimentally constrained. Their complexity prevents analytical treatments, so that most such models are formulated in terms of agent-based or cellular automata approaches. As a result of this complexity, very few of these models have provided analytical insights, or have been compared to experiments quantitatively. In summary, we are not aware of 3-d models of colony growth that account for the spatially varying density of nutrients and bacteria, explain the observed experimental phenomenology of bacterial growth in such colonies, and do so in a relatively simple coarse-grained (PDE) Monod-style manner, rather than relying on complex agent-based simulations of individual bacteria.

**Fig 1:**
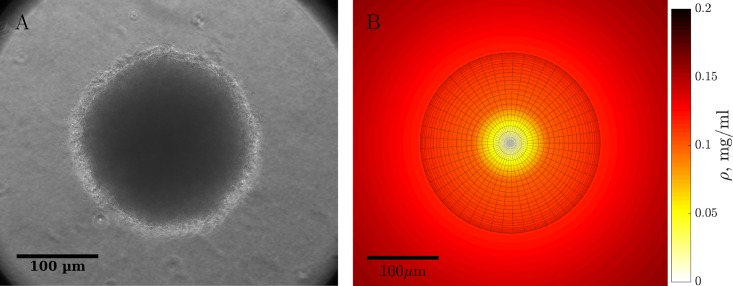
3-d colony growth. (A) Photograph of a representative *E. coli* colony inside 3-d agar at 22 hrs post inoculation. (B) A growing colony at 22 hrs as simulated using our mathematical model. Heatmap shows the spherically symmetric nutrient concentration, and the meshgrid sphere represents the colony. At this time, the nutrient at the center of the colony is fully consumed. Since the growth rate depends on local nutrient concentration, the cells at the center of the colony are not growing anymore.

Here we develop such a model that treats the growth rate heterogeneity due to the non-uniform nutrient distribution produced self-consistently by consumption of a nutrient by the bacteria. We explore its fit experimentally with the growth of *E. coli* maintained and growing as colonies embedded in 3-d matrix of soft agar with an initially uniform spatial distribution of a limiting carbon source, glucose (Fig 1). We compare dynamics of growth of bacteria in colonies with that of planktonic cells in liquid culture with the same concentration of limiting glucose. In our model, we assume that the colony is essentially unconstrained by the soft agar and is free to expand, and the bacteria within it are non-motile. This combined theoretical-experimental study reveals two surprising features of bacterial populations growing as colonies: (i) the bacteria within these structures exist as loosely packed viable cells, and (ii) the viable cell densities of bacteria produced in colonies is more than two-fold greater than that in liquid cultures with the same concentration of the limiting glucose.

## Results

### Experimental characterization of bacterial growth

We use population growth of *E. coli* in minimal medium as the basis for developing the model. To control the amount of nutrients available to the bacteria, we use glucose at the initial concentration 0.2 mg/ml, at which it limits the stationary phase density of *E. coli* produced as planktonic cells and as colonies in soft agar. We grow bacteria either in liquid cultures or as three-dimensional colonies embedded in soft agar (Fig 1). Unless otherwise noted, 3-d colonies are grown in 3 ml of soft agar, inoculated with approximately 50 bacteria/ml. Under these conditions, each colony has an access to a nutrient subvolume of *v* ∼ 1/50 ml, or, on average, a nutrient sphere of radius *R* = (3*v*/4*π*)^1/3^ ≈ 1.7 mm. For the liquid and the 3-d growth, we estimate the density of viable cells, *N*(*t*), at different times diluting and plating and then counting the number of resulting colonies (colony-forming units, or CFU), see Methods for details. For each time point, we obtain 6 independent replicates of CFU density estimates, and each experiment was replicated at least 3 times.

**Fig 2:**
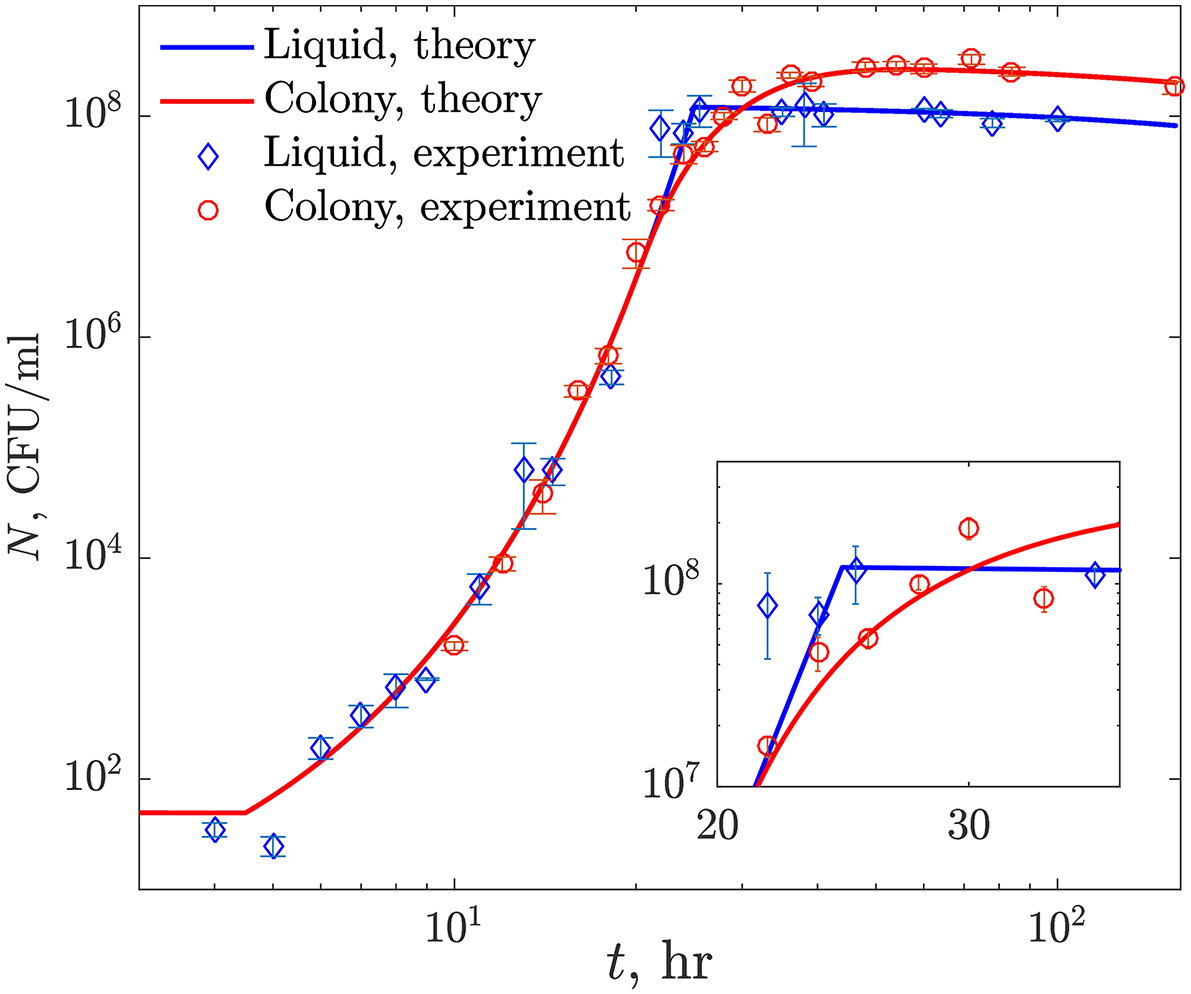
*E. coli* population dynamics. Experimental data, averaged over all experiments (symbols, error bars are s. e. m.), are compared with the fits of the mathematical model we developed (solid lines). For clarity, uncertainty of the numerical predictions is omitted and is shown instead in Fig 4. Liquid cultures switch abruptly from the exponential growth to the saturation, and then decay slowly. In contrast, 3-d colonies gradually slow down before saturating (see Inset) at a population size larger than that in the liquid, and then decay. Note that the curves start with ∼50 CFU/ml, which corresponds to over ∼150 colonies started by individual cells in the 3-d colony setup.

The results of these population growth experiments are shown in Fig 2 (data points). In liquid, the density of the population increases exponentially, and then abruptly stops and begins to decline at a low rate, presumably because the bacteria consume the available glucose and enter the stationary phase, at which time the rate of cell mortality exceeds that of division. In contrast, in 3-d colonies, the exponential growth and the stationary phase are separated by a gradual decline in the net rate of growth. We expect that this is because the growth of the population here is limited by the speed with which diffusion brings glucose from the periphery of the available nutrient volume to the colony, where it is consumed by the bacteria. Surprisingly, the maximum density of bacteria growing as colonies is substantially greater than that in liquid, despite the concentration of the limiting glucose being equal for liquid and the soft agar. To understand these findings quantitatively, we now develop a simple (minimalist) mathematical model of resource-limited bacterial growth in liquid and as spatially structured colonies.

### Minimal model of resource-limited bacterial growth

Our liquid culture model of bacterial growth is a variant of that of Monod [2]. In this model, all bacteria have the same resource (glucose) concentration dependent growth rate *g*(*ρ*) = *g*_max_ *ρ*/(*ρ* + *K*), where *ρ* is the concentration of glucose, *g*_max_ is the maximum growth rate, and *K*, the Monod constant, is the concentration of the resource when the growth rate is half its maximum value *g*_max_/2. With these parameters, the rate of change of the density of the bacterial population *n* = *N*/*v* is given by

$$\begin{array}{cc} \frac{dn}{dt} & =n\;\Theta \left(t-\tau_{\text{lag}}\right)\frac{g_{\text{max}}\;\rho }{\rho +K}-nm, \end{array}$$ (1)

$$\begin{array}{cc} \frac{d\rho }{dt} & =-\frac{1}{a_{l}}n\;\Theta \left(t-\tau_{\text{lag}}\right)\frac{g_{\text{max}}\;\rho }{\rho +K}. \end{array}$$ (2)

Here *v* is the volume of the liquid where the culture grows, and *a*_l_ is the liquid *yield*, which measures the number of bacteria produced by a microgram of the nutrient. Further, Θ(*t* − *τ*_lag_) is the Heaviside Θ-function, which is equal to zero for *t* < *τ*_lag_, and to unity otherwise. It represents the lag phase before the growth starts after a transfer to a new environment. Note that Eqs (1) and (2) differ slightly from the standard Monod model. Specifically, we added a small constant death rate *m* to account for the decrease of the population in the liquid culture after the saturation (Fig 2). Thus the population has a zero net growth at a critical nutrient density of *ρ*_m_ = *mK*/(*g*_max_ − *m*), which represents the minimum nutrient concentration needed to sustain life without growth [27].

We fit the five growth parameters (*g*_max_, *K*, *a*_l_, *τ*_lag_, and *m*) to the experimental data using nonlinear least squares fitting, and estimate uncertainties of the fit using bootstrapping (see Materials and methods, and also Table 1). As seen in Fig 2 (blue curve), after the lag phase, the population increases exponentially before it saturates abruptly when all the cells in the colony run out of food at the same time. The excellent agreement between the experiments and the model is encouraging. It allows us to use the Monod model with death as the basis for 3-d studies.

**Table 1:** Fitted parameters.

Name	Description	Literature values	References	Fitted value	80% confidence interval
*g*_max_	maximum growth rate, hr^−1^	[0.52, 0.83]	[32, 36, 37]	0.73	[0.56, 0.89]
*K*	half-saturation constant, *μ*g/L	35	[37]	122	[19.5, 783]
*a*_l_	yield in liquid, 10^6^ CFU/*μ*g glucose	[0.5, 1.2]	[38, 39]	0.61	[0.54, 0.67]
*τ*_lag_	lag phase duration, hr	[2, 5]	[40]	4.5	[4.3, 4.7]
*m*	death rate, hr^−1^	[0.0049, 0.018]	[27, 37]	0.0029	[7.7 × 10^−4^, 0.011]
*a*_c_	yield in 3-d colony, 10^6^ CFU/*μ*g glucose	N/A		1.50	[1.36, 1.63]
*D*	glucose diffusion in 0.35% agar, *μ*m^2^/hr	1.8 × 10^6^	[41]	0.55 × 10^6^	[0.21, 0.89] × 10^6^
*μ*	packing density, CFUs/*μ*m^3^	N/A		2.98 × 10^−2^	[1.74, 4.22] × 10^−2^

To develop the minimal model of 3-d growth, we assume that the bacteria within the colony are physiologically identical, but depending on their position, vary in their access to the diffusing carbon source. Thus all cells grow according to the Monod model, differing only by the local availability of the limiting nutrient, glucose, *ρ*(*x*, *y*, *z*). For 3-d colonies, the dynamics of the bacterial density *n*(*x*, *y*, *z*) is a result of a complex interplay between mechanical properties of the extracellular colony matrix and the substrate, in which the colony grows, the stiffness of the bacterial wall, and the growth properties of bacteria themselves. Mathematical models that account for these complexities are typically over-parameterized, making it hard to make precise quantitative predictions [23, 24]. In contrast, here we aim at building the simplest possible model consistent with data. We assume that soft agar is too soft to provide mechanical resistance to the colony, but sufficiently dense to keep cells from moving. Thus the colony would grow in density until bacteria get as close-packed, on average, as possible given the amount of the extracellular matrix they secrete. We denote this maximum cell number density as *μ*. Having reached the maximum density, any new cellular divisions must expand the colony into the soft agar, with the same fixed maximum density in the volume occupied by the colony, save for possibly smaller density at the colony edge. Further, as seen in Fig 1, the colony spreads spherically-symmetrically, so that the density of the cells and the nutrient concentration are functions of the radius and the time only, *n*(*r*, *t*) and *ρ*(*r*, *t*). Thus we have

$$\begin{array}{cc} \frac{\partial n(r,t)}{\partial t} & =n\left(r,t\right)\;[\Theta \left(t-\tau_{\text{lag}}\right)\frac{g_{\text{max}}\;\rho \left(r,t\right)}{\rho (r,t)+K}-m], \end{array}$$ (3)

$$\begin{array}{cc} \frac{\partial \rho (r,t)}{\partial t} & =D\nabla^{2}\rho -\frac{1}{a_{c}}n\left(r,t\right)\;\Theta \left(t-\tau_{\text{lag}}\right)\frac{g_{\text{max}}\;\rho \left(r,t\right)}{\rho (r,t)+K}, \end{array}$$ (4)

with the initial uniform spatial concentration of the nutrient *ρ*(*r*, 0) = *ρ*_0_ at time *t* = 0, and a single bacterium starting the colony at *r* = 0. In these equations, *D* is the nutrient (glucose) diffusion coefficient. Further, we allow for the yield in the colony *a*_c_ to be different from the liquid yield *a*_l_ to account for the different saturation values in Fig 2, as further discussed below. Importantly, since the agar is more than 99% liquid, the four other growth parameters *g*_max_, *K*, *τ*_lag_, and *m* are taken to be the same in both media.

Bacterial density in Eq (3) would grow to infinity with time, which is physically unrealistic. Thus we need to bound the cell density from above by a maximum value, corresponding to maximally packed cells (and extracellular matrix) that can be compressed no further. To do this, and to establish such maximum cellular packing density *μ* in Eq (3), we now impose that the overall increase in cell number beyond the maximum density leads to the proportionate growth of the colony radius *r*_c_, so that $N\equiv 4\pi \int dr\;r^{2}n\left(r,t\right)=\left(4/3\right)\pi r_{c}^{3}\mu$. In other words, at each point in time, we impose the condition that

$$\begin{array}{c} n\left(r,t\right)=\{\begin{array}{cc} \mu , & 0<r\leq r_{c}={\left(3N/4\pi \mu \right)}^{1/3},\\ 0, & r_{c}<r\leq R, \end{array} \end{array}$$ (5)

where *R* = (3*v*/4*π*)^1/3^ is the radius of the nutrient subvolume accessible to the colony. To reconcile Eqs (3) and (5), we say that all new growth is accounted for by the expansion of the colony edge, *r*_c_(*t*), while the death results in a decrease in the cell density locally (see Materials and methods for description of the algorithm for simulating this growth model). This is reminiscent of earlier hybrid differential-discrete simulations [28, 29]. However, we note that, in our model, the biomass changes differentially: the cell density in each spherical shell is a real number, and it is not necessarily equal to *μ* in either the largest shell (due to growth) or in all other shells (due to cell death). In fact, we emphasize that one should not view the colony growth as biomass transfer, but rather as growth in the inner shells creating pressure that expands the colony radius continuously. It might be possible to write this mass dynamics as an integro-differential equation. However, for the purpose of solving the model and comparing to experiments, an integro-differential equation will not be more useful than the explicit dynamical rules that we have provided.

Although the assumption of redistribution of new growth into a spherically symmetric front of a growing colony will likely be violated for a generic colony or biofilm, it is clearly satisfied for colonies in our experiments (cf. Fig 1). One would expect violations of the symmetry if there are nearby colonies competing for the same nutrients, and thus partially shielding each other. However, in our experiments, colonies are either well separated and hence weakly interacting (small initial bacterial densities), or there are many colonies in arbitrary directions from each other (large bacterial densities); in both cases, the approximate spherical symmetry is restored (especially since we average over multiple colonies before counting CFUs). Thus there are no obvious reasons to go beyond the spherical symmetry assumption. In fact, we will see that predictions of this simple model will be verified against new experimental data, further confirming the spherical symmetry assumption *a posteriori*.

As mentioned above, there are a lot of models in the literature describing growth of bacterial communities in spatially structured environments. [19, 23, 24]. However, we have not found any readily available 3-d spherically symmetric soft agar colony models, where every included biological process or physical feature is essential to the population biology of the colony. This necessitated development of our model in this section.

### Model analysis

**Fig 3:**
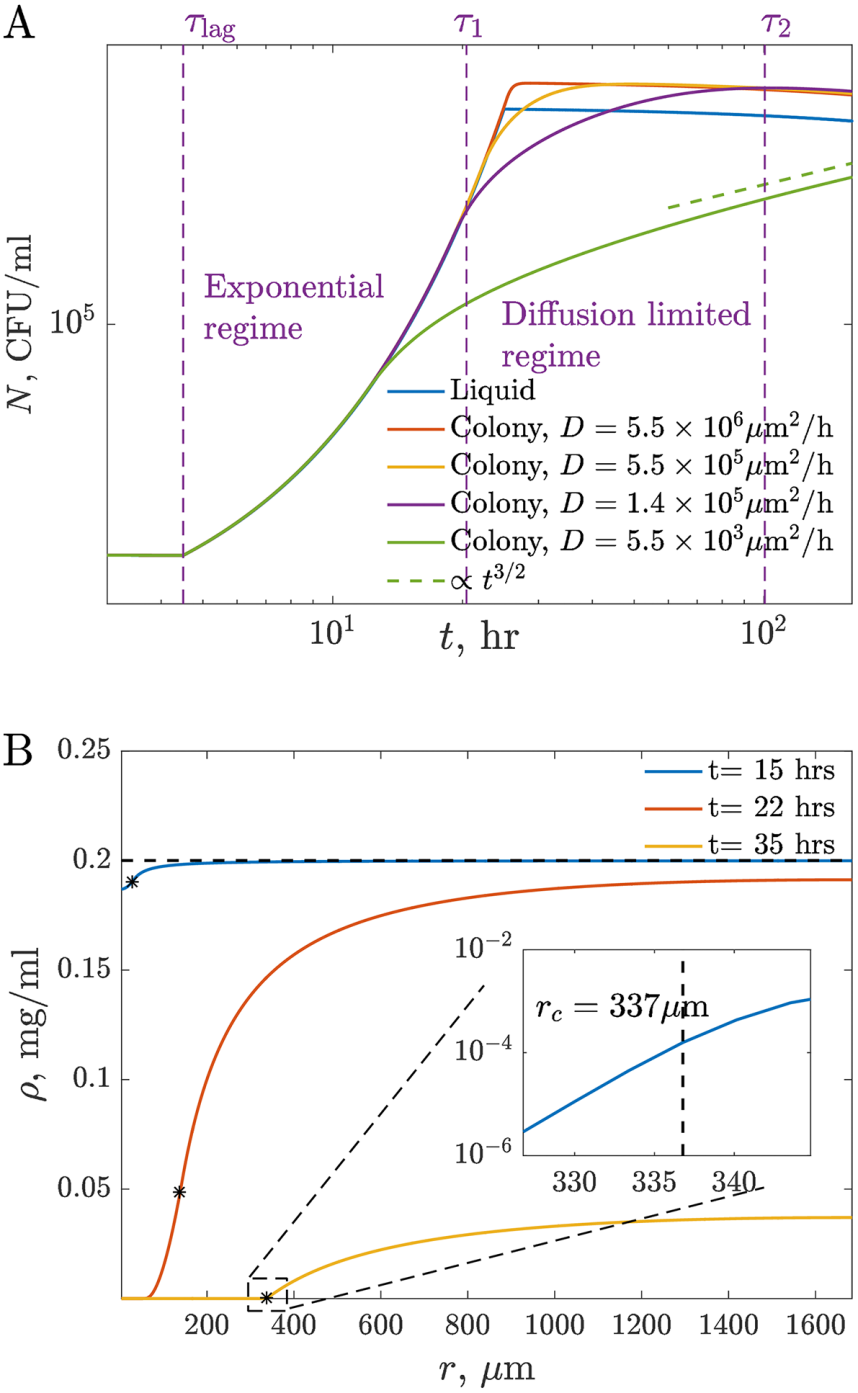
Mathematical model predictions. (A) Population growth in liquid culture and in 3-d colonies. The growth parameters are chosen as best fit values for our experimental data (see Table 1), except for *D*, which we vary to illustrate different growth regimes. The diffusion-limited regime in the limit of small *D* is consistent with the prediction *N* ∝ *t*^3/2^. The time scales *τ*_*i*_ are illustrated for *D* = 1.4 × 10^5^ *μ*m^2^/hr. (B) Profile of the nutrient concentration in space at different times using the same parameters as above and *D* = 5.5 × 10^5^ *μ*m^2^/h, as in Table 1. The edge of the colony is illustrated by stars on each curve. The inset shows that the concentration decreases exponentially at the colony edge in the diffusion-limited growth regime. The penetration depth is about 3 *μ*m.

To illustrate the behavior of the 3-d model of bacterial growth as colonies, we plot numerical solutions of Eqs (3)–(5) for different values of the nutrient diffusion coefficient in Fig 3(A). Especially at small *D*, two different growth regimes are clearly visible after the lag but before the ultimate saturation and the slow cell death. The first is the fast *exponential growth* based on local, immediately accessible resources. This regime is indistinguishable from the growth in liquid. When the local nutrients are depleted at a certain time *τ*_1_ following the start of the growth at *τ*_lag_, new nutrients must be brought from afar by diffusion. This is slow, resulting in a slower *diffusion-limited growth* regime. Here the overall colony growth rate is an average over cells growing at different rates due to different concentrations of the locally accessible nutrient. Our numerical solutions suggest that, in this regime, the nutrient concentration at the colony edge decays exponentially fast, in agreement with Ref. [18], cf. Fig 3(B). The nutrient penetration depth is only a few *μ*m, or a few cell layers. Therefore, in the diffusion-limited regime, there are, essentially, no nutrients deep inside a colony, and only cells at the periphery can grow. In the absence of resource storage [30], nutrient sharing from the outer cells, or cannibalism (we model none of these), interior cells would not grow at all and will eventually die. The diffusion-limited growth regime finally ends with *saturation* and *slow death* when most of the nutrients in the accessible subvolume are depleted at time *τ*_2_ after *τ*_lag_. The onset of the saturation takes longer than in liquid since small (but larger than *ρ*_m_) amounts of the nutrient linger at the far edges of the nutrient subvolume for a long time.

Analytical expressions for *τ*_1_, *τ*_2_, and the growth dynamics can be obtained from the following arguments. First, in the exponential growth regime, the population grows as $N∼e^{g_{\text{max}}t}$. This requires $e^{g_{\text{max}}t}/a_{c}$ of the nutrient mass, which must come from the volume immediately accessible by diffusion, equal to $∼\rho_{0}{\left(\sqrt{Dt}\right)}^{3}$. Equating the two expressions gives, to the leading order, $\tau_{1}∼g_{\text{max}}^{-1}\log\left[\rho_{0}a_{c}{\left(D/g_{\text{max}}\right)}^{3/2}\right]$. When local resources are exhausted, growth is limited by nutrients diffusing in from the volume $∼{\left(\sqrt{Dt}\right)}^{3}$. However, because the encounter probability for a 3-d random walk is less than one [31], most of the nutrient molecules coming from afar will not be immediately absorbed. In fact, since the box-counting dimension of a diffusive process is two, only $∼\rho_{0}{\left(\sqrt{Dt}\right)}^{2}r_{c}$ nutrient molecules will be captured in time *t*, resulting in *N* ∼ *ρ*_0_ *Dtr*_c_ *a*_c_. On the other hand, the radius of the colony grows as *r*_c_ = (3/4*π*)^1/3^(*N*/*μ*)^1/3^. Combining these expressions gives *N* ∼ [(*a*_c_ *ρ*_0_ *D*)^3^/*μ*]^1/2^ *t*^3/2^ in the diffusion-limited regime. Finally, the total amount of nutrients available to the colony is ∼*ρ*_0_ *R*^3^, and so the diffusion-limited growth will saturate, and the cells will start dying with the rate of *m* when the colony grows to *N* ∼ *a*_c_ *ρ*_0_ *R*^3^, which occurs at *τ*_2_ ∼ (*μ*/*a*_c_ *ρ*_0_)^1/3^ *R*^2^/*D*. Altogether, we find

$$N∼\{\begin{array}{ll} \text{const}, & t<\tau_{\text{lag}},\\ e^{g_{\text{max}}t}, & t-\tau_{\text{lag}}\ll \tau_{1}∼\frac{\text{log}\left[\rho_{0}a_{\text{c}}{\left(\frac{D}{g_{\text{max}}}\right)}^{3/2}\right]}{g_{\text{max}}},\\ {\left[\frac{{\left(a_{\text{c}}\rho_{0}D\right)}^{3}}{\mu }\right]}^{\frac{1}{2}}t^{3/2}, & \tau_{1}\ll t-\tau_{\text{lag}}\ll \tau_{2}∼{\left(\frac{\mu }{a_{\text{c}}\rho_{0}}\right)}^{\frac{1}{3}}\frac{R^{2}}{D},\\ a_{\text{c}}\rho_{0}R^{3}e^{-mt}, & \tau_{2}\ll t-\tau_{\text{lag}}, \end{array}$$ (6)

These analytical estimates are supported by the numerical solutions in Fig 3(A).

We note that in one or two dimensions, the diffusion limited growth would scale as *N* ∝ *t*^*d*/2^ for dimension *d*, independently of the (small) colony radius, or even for a point colony, since the random walk encounter probability there is one [31]. In contrast, our three-dimensional result depends critically on knowing how the radius of the colony scales with the number of growing bacteria. In particular, here we cannot model the colony as a point-like object. Thus the exponent of the power law scaling is not universal in 3-d, and it may change for heterogeneous colonies with varying cell size and cell density.

### Comparing the minimal model of 3-d bacterial colony growth to data

**Fig 4:**
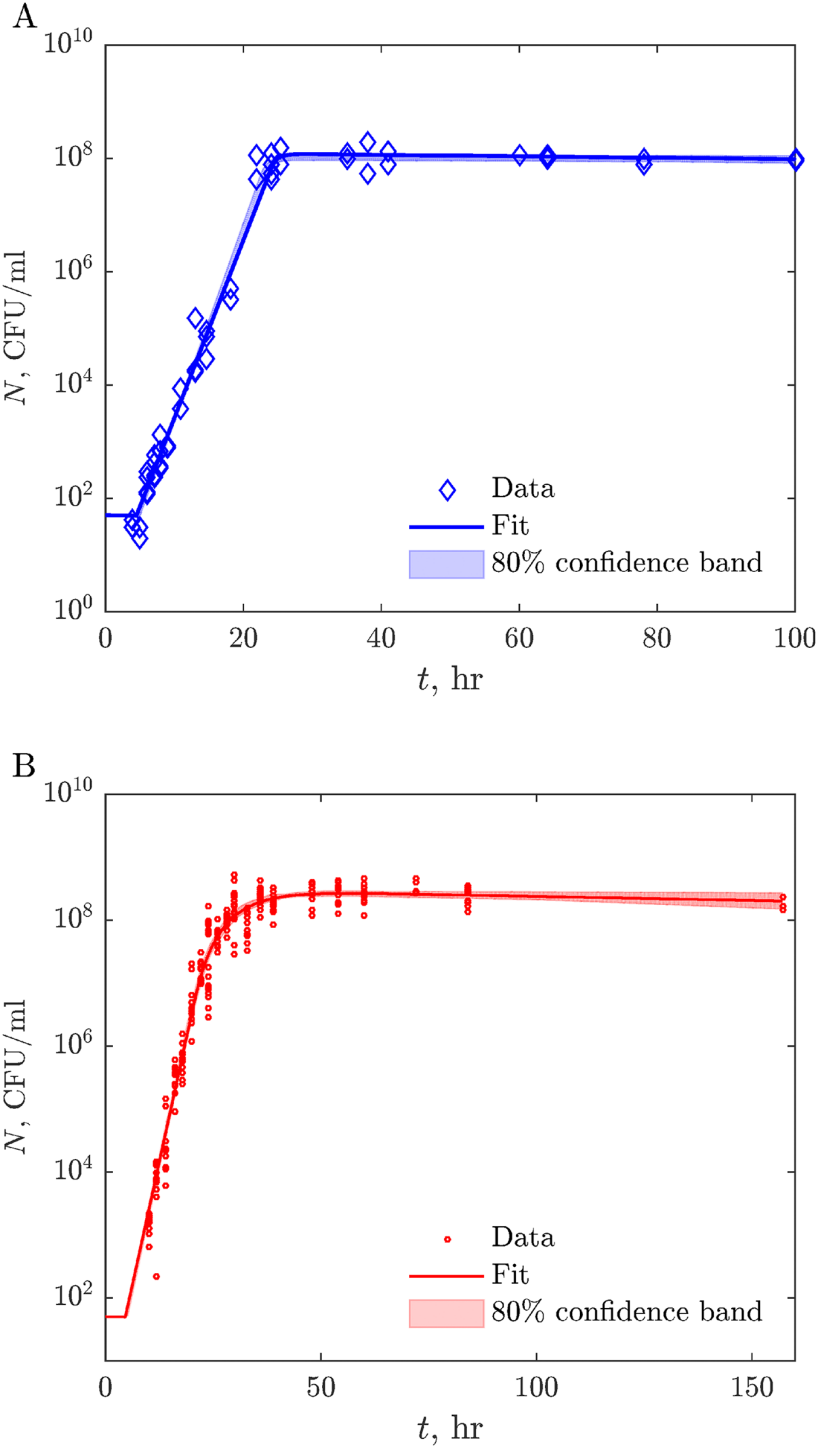
Fitting models to data. *A*: Liquid growth model (solid blue line) fitted to all of the experimental data we have collected (blue diamonds). 80% confidence intervals around the best-fit predictions are shown by light blue shaded bands (established by bootstrapping, with 1000 resamplings). *B*: same, but for 3-d colony growth. Red circles, solid red line, and light red band correspond to the data, the best fit, and the 80% confidence intervals (from 30 resamplings).

To determine the extent to which our minimal model accounts for the dynamics of growth of bacteria in colonies, we fit the model to data using nonlinear least squares fitting, similar to the liquid case. We keep the parameters *a*_l_, *K*, *g*_max_, *m*, and *τ*_lag_ equal to the values inferred for liquid, and only optimize *D*, *μ*, and *a*_c_ for the 3-d culture data. See Materials and methods for the details of the fits, including estimation of the prediction uncertainty using bootstrapping. Table 1 shows fitted parameter values with the corresponding nominal values from the literature. The fitted parameters are consistent with the nominal values where the latter are known. A possible exception is the value of the glucose diffusion coefficient *D*, which is lower than those reported in previous publications (though the confidence interval on our fits is rather large). This could be a result of the previous measurements done in hydrogels, rather than in 0.35% agar preparation used in this study. Further, the best fit curve shows an excellent agreement with data (cf. Fig 2, red), and the prediction confidence bands are very narrow (cf. Fig 4). This suggests that nutrient diffusion and the ensuing geometric heterogeneity of growth are sufficient to explain the population dynamics of these *E. coli* colonies in 3-d at our experimental precision, and consideration of additional phenotypic inhomogeneities is not needed.

Our analysis also provides estimates of two previously unknown parameters, *μ* (the maximum packing density of viable cells) and *a*_c_ (yield in 3-d colonies). The inferred packing density is *μ* = 3.0 ⋅ 10^−2^ CFU/*μ*m^3^, with the 80% confidence interval of [1.7, 4.2] ⋅ 10^−2^ CFU/*μ*m^3^. Since an *E. coli* cell has a volume of between 0.5 and 2 *μ*m^3^ [32, 33], this suggests that only about ∼3% of all space in a colony is occupied by viable cells. This is a surprising finding, and it requires an independent corroboration. Towards this end, we measure radii of large colonies and calculate their packing densities by diving colony volumes by the average CFUs per colony. This gives *μ* = 1.5 ± 0.08 × 10^−2^ CFU/*μ*m^3^, consistent with our estimation of *μ* from the fitted growth model. In other words, in our experiments, viable *E. coli* cells are sparsely packed. Notice that here we only say that, in large colonies, there is a small density of *viable* cells that grow into visible, countable colonies when plated. There could be many other cells, which, for whatever reason, do not grow into large colonies after plating. Without additional investigations, we cannot make the distinction. Also notice that we can only make this claim for *large, old* colonies which is when most cells in growing colonies emerge. In particular, the low density claim is not valid during early growth, when each colony starts with an individual cell, which by definition takes 100% of the colony volume.

The second inferred parameter is *a*_c_. We find that the yield as measured by the ratio of the CFU estimated stationary phase density and the quantity of glucose in 3-d is 2 to 3 times higher than that in liquid culture, *a*_c_ > *a*_l_ (cf. Table 1). This implies that, at saturation, colonies produce more CFUs than liquid cultures, which is directly apparent from Fig 2. This is a surprising result, since in the colony the bacteria grow more slowly and there is more time for cell death. Nonetheless, similar results have been reported for colonies growing on surfaces [34]. Here this effect is likely a direct consequence of the growth dynamics during the diffusion-limited regime. Indeed, *E. coli* cells growing at a rate of >1 hr^−1^ grow to be 2 to 3 times larger than cells growing at a rate of <0.1 hr^−1^ [35]. While the diffusion limited regime lasts only for a few hours (cf. Fig 2), more than 90% of all cells emerge at that time, so that the majority of cells in the colony are smaller than in liquid, yielding more cells from the same nutrient amount.

### Independent experimental test of the model

**Fig 5:**
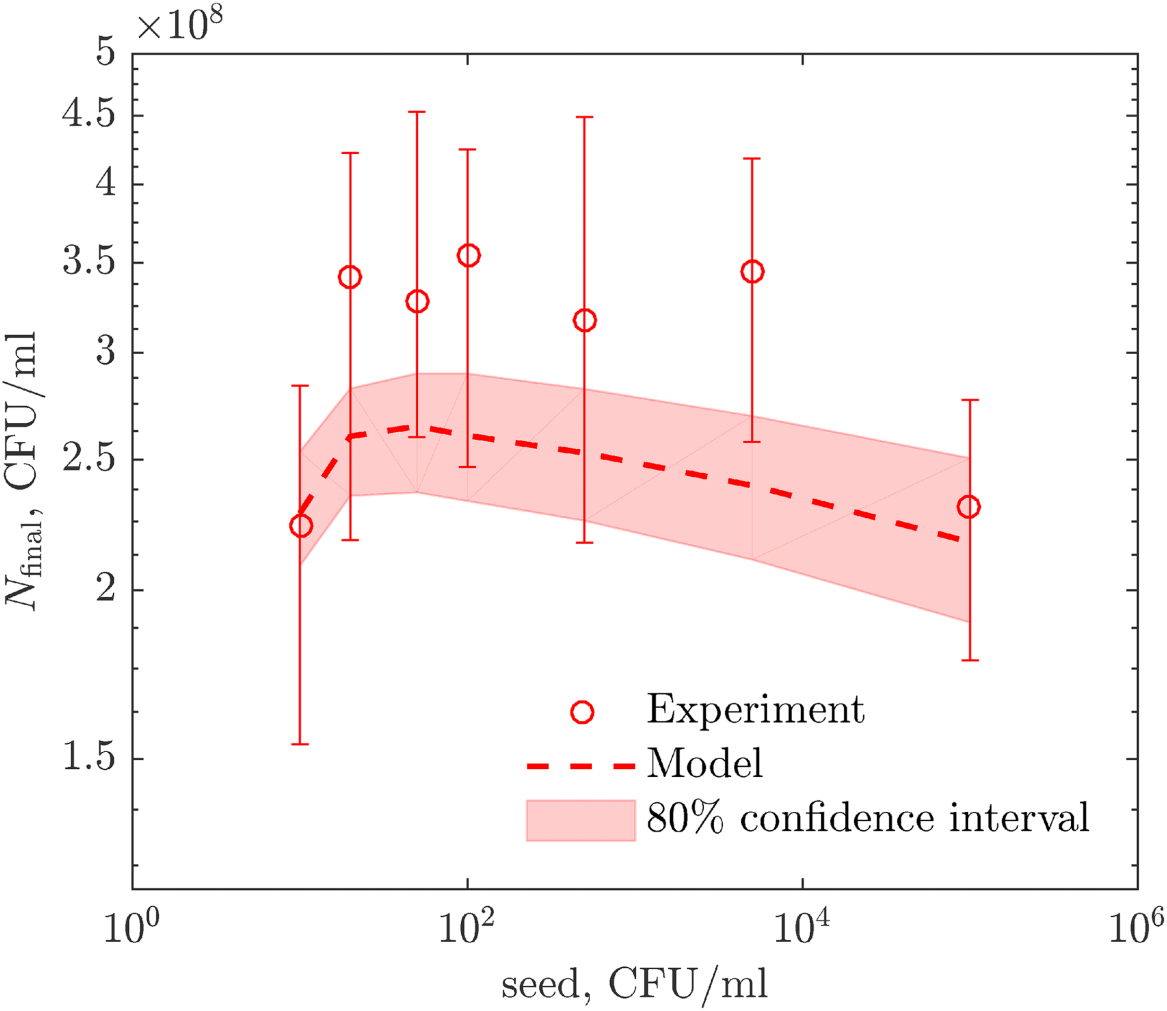
Dependence of the population size on the inoculation density. Colony cultures inoculated with different cell densities grow to different population sizes. Circles are experiment data measured at 74 hr post inoculation, and error bars are s. e. m. The best-fit 3-d bacterial growth model reproduces these data within experimental error bars and computational confidence interval, without additional fitting.

As an independent test of the developed 3-d growth model, we use it to predict results of experiments distinct from those used for fitting the model. Specifically, we investigate how the population size depends on the density of bacteria used to inoculate the soft agar. At a long measurement time (72 hrs), our model predicts a non-monotonic dependence of the population size on the inoculation density (cf. Fig 5, dashed line). This is because, at very low densities, each colony has access to a large nutrient subvolume, and the colony cannot clear this subvolume by diffusion in just 72 hrs. As a result, at the end of the experiment, there are still nutrients in the media, and the colony does not reach its maximum size. In contrast, at very high inoculating densities, colonies rapidly exhaust their small available nutrient subvolumes, the cell death becomes important throughout much of the experiment duration, and the population is smaller again. Thus the population reaches its maximum at intermediate densities, where these two effects balance. We test this prediction by experimentally measuring population sizes at 72 hrs for *E. coli* growing in soft agar at inoculums varying from 10^1^ to 10^5^ cells/ml As seen in Fig 5, the experimental data agree with the prediction within errors and, in particular, exhibit the expected non-monotonicity. We emphasize that no additional fitting was done for this figure, and yet the agreement between the experiment and the theory is very good.

### Experimental test beyond population biology confirms prediction and suggests future model improvements

Our simplest 3-d spherically symmetric colony growth model has been able to fit bacterial population dynamics data remarkably well. To further challenge the model and to suggest its possible future improvements, we now use microscopy (see Materials and methods) to collect additional data that is of a very different nature compared to the data used for building the model. We use these new experiments to further investigate the most salient prediction of the model, namely the difference in the liquid vs. colony yield.

**Fig 6:**
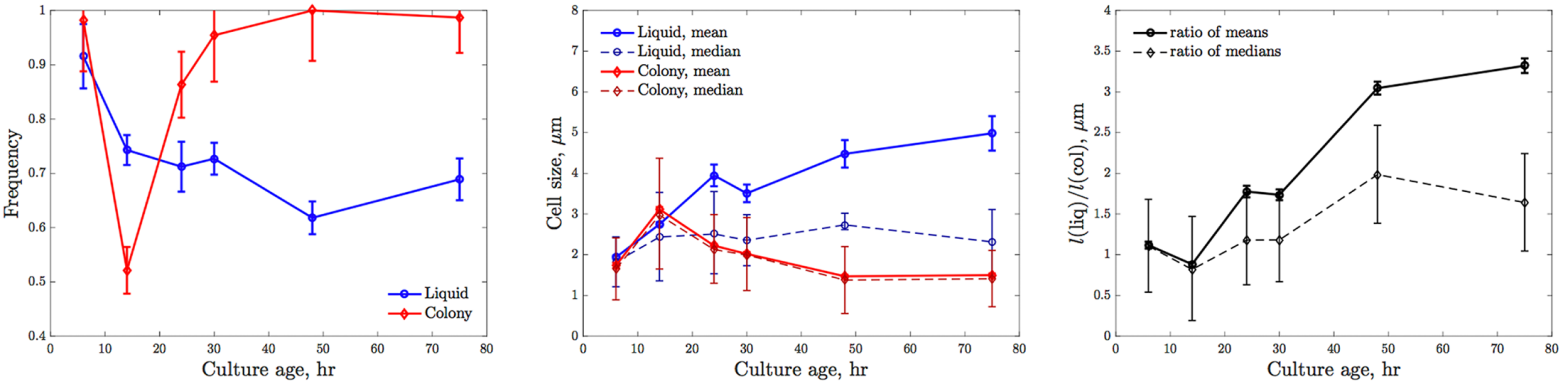
Cells in liquid and in colonies have different sizes. (Left) The fraction of non-filamentous cells (< 5 *μ*m in length) in liquid cultures and in colonies. Color convention is as in the previous figures. Error bars represent the square-root counting statistics. (Center) The mean and the median cell sizes in liquid and colony cultures. Error bars of the means are s. e. m. Error bars of the medians are the bootstrapped 95% confidence intervals. (Right) The ratio of cell sizes in liquid to those in colonies. The solid line shows the ratio of the means, and the dashed line is the ratio of the medians. Error bars are propagated from the error bars of the means and the medians. Cells in old colonies are 1.6 to 3.4 times shorter than in old liquid cultures.

According to our fits, the bacterial yield per microgram of glucose in 3-d colonies is 2 to 3 times higher than that in the liquid culture. We proposed that this is because the cells in liquid grow faster (and hence are larger) than those in colonies. To verify this directly, we measured the cell size (length) in these different growth conditions as a function of the time since inoculation (see Materials and methods). For liquid, the mean cell length at 6 hours post-inoculation was 1.9 ± 0.7 *μ*m. For older cultures, we have no way of distinguishing young and old cells, and so the distribution of cell sizes includes both cells that were born in earlier stages of the experiment, as well as recently. Crucially, as the cultures grew older, long, filamentous cells emerged (for old cultures, the longest cells were > 100 *μ*m). While the fraction of such extremely long cells was small, this tail of the cell size probability distribution [42, 43] had a pronounced effect on the mean cell length, increasing it to ∼5 *μ*m for the oldest cultures (Fig 6). To account for this long tail, we report both the mean and the median cell sizes, as well as the fraction of cells that remained non-filamentous (defined as < 5 *μ*m in length). As seen in Fig 6, the number of short cells stabilizes near 60 − 70% for the oldest cultures. At the same time, the median cell size in liquid does not depend on the culture age, hovering around 2 *μ*m. In contrast, the distribution of cell sizes in colonies is much less skewed. Less than 1% of the sampled cells become filamentous at long times (Fig 6), so that the mean and the median cell lengths are nearly equal. The average cell size drops when the diffusion-limited growth starts, and it saturates near 1.5 *μ*m for very old colonies. Combining these measurements, the size ratio of cells grown in the liquid and in colonies is between ∼1.6 (for the median length) and ∼3.4 (for the mean length), in agreement with the population biology estimate above, again validating our model. Crucially, these experiments also suggest that the immediate next modification of our growth model should not be inclusion of mechanical stresses and more complicated nutrient and waste product fluxes, but rather the growth-speed dependence of the yield, *a* = *a*(*g*(*ρ*)), which would replace the two parameters *a*_l_ and *a*_c_ with a single function and unite the two growth models.

## Discussion

To our knowledge, the model developed here is the first continuous, rather than agent-based, model to explicitly study bacterial growth as colonies. We consider this the minimal model because it assumes spherical symmetry and that the availability of nutrients (a carbon source) is the sole factor determining the rate of cell division within colonies. In reality, the cellular growth, division, and death rates would also depend on cell-to-cell interactions of various sorts, on the enrichment and deterioration of the environment due to the buildup of secondary metabolites and waste, on cell-environment mechanical interactions, and on diverse cellular phenotypic commitments. The model we developed and experimentally tested here only accounts for the spatial heterogeneity in access to the diffusing nutrient and assumes no such additional effects [30, 44, 45].

Nevertheless, despite these limitations, with five parameters describing the growth in liquid, and three additional parameters specific to the 3-d spherically symmetric colony growth, this model provides an impressively accurate description of growth of populations of *E. coli* as colonies in soft agar as well as planktonic cells in liquid. Unlike the anticipated and observed nearly precipitous termination of growth in liquid culture as nutrients become depleted, our 3-d model accounts for the experimentally observed gradual reduction in net rate of replication as diffusion of the resource increasingly limits colony growth with time. With no additional fitting, the model also correctly predicted the non-monotonic, upside-down U shaped dependence of the population size on the inoculating bacterial density. Moreover, all of the best-fit parameters inferred from the data agreed with prior estimates in the literature, where these are available (see Table 1 and references therein), indicating high-quality fits without overfitting.

Our study has revealed and/or confirmed several intriguing observations about bacteria growing in colonies. First, the growth in colonies yielded substantially greater viable cell densities than obtained in liquid culture with the same concentrations of limiting carbon source. We proposed that this was a direct consequence of the diffusion-limited growth, which happens at a slower division rate. In turn, slow division is correlated with smaller size of bacterial cells [35], resulting in more bacteria for the same nutrient amount. This slowing down is very important phenotypically—according to our model, over 90% of all bacteria in the colony are formed at such decreased growth rate, and the yield *a*_c_ is an average over yields at different stages of the slowing. We have directly verified this hypothesis by measuring cell sizes in liquid and colony cultures as a function of the time since inoculation. These new data provided a confirmation of our population biology estimates of the yields and of the hypothesis behind their difference. The experiments also suggest a natural future extension of our model, which would come from measuring the dependence of the cell size and the yield on the growth rate and then verifying if both the liquid and the colony growth can be described by the same dependence *a* = *a*(*g*(*ρ*)).

Our second intriguing observation, which is supported by two independent sets of measurements, is that the packing density inside colonies is very low, *μ* ∼ 0.03 CFU/*μ*m^3^, so that the vast majority of a volume of a colony is not occupied by viable cells. The accuracy of this observation depends strongly on whether, when the 3-d cultures are liquified and plated for counting, cells get perfectly separated from each other, and the counted colonies start from individual cells. We notice that, when we put liquified cultures under a microscope to produce data for Fig 6, we observe that the cells *are* well-separated. Further, if low *μ* was a result of us underestimating the number of cells in the colony, it would mean that *a*_c_ must be even larger than our current estimate. Thus one would be able to make even more cells from the same nutrient amount in 3-d cultures. This is extremely unlikely since we additionally measured the cell lengths in liquid and in 3-d colonies (Fig 6), with the ratio of cell lengths agreeing with the independently estimated ratio *a*_l_/*a*_c_. Thus we are confident in our estimate of *μ*. What could be the reasons for its small value? It is possible that the colonies are, indeed, largely void of viable cells, with extracellular fluids and matrix fibers filling in the gaps. Another possibility is that cells deep inside the colony are dead or dormant due to the absence of nutrients, or due to other effects, such as mechanical stresses, so that the viable cells that we measure are a minority of all the bacterial cells that existed. Our experiments show no evidence for such deviations from the minimal growth model, but it is clear that additional studies, including direct imaging of the colony structure, must be done in the future.

One interpretation of the close fit between the predictions of this minimal model and the results of our soft-agar experiments is that heterogeneities beyond nutrient access contribute little to the growth dynamics of bacteria in colonies. It remains to be tested how general this result is. Is the *E. coli* in glucose-limited minimal medium used in this experiment exceptional? Is the spherically symmetric growth special? Will the results hold for other bacterial species and for complex media, like broth? We propose that the minimal model developed here be used as a baseline to address such questions of generality with other bacteria, geometries, and media. Models are most useful when they do not fit data and thus point to other factors contributing to the studied dynamics. For growth of bacteria in colonies, such factors can be mechanical or other stresses, cell-cell interactions, and others. From an evolutionary perspective particularly intriguing would be studies of growth of bacteria in colonies initiated with multiple cells of different genotypes (or even species), where deviations from the model could signal such important phenomena as clonal competition or cooperation within a clone.

## Materials and methods

### Bacteria

We used *Escherichia coli B, ara rpsL T6 r-m-*, originally obtained from Seymour Lederberg [38]. We employ one of the evolved strains, REL1976, generously provided by Richard Lenski [46].

### Media, culture and sampling procedures

Overnight cultures were grown in Lysogeny Broth (LB), Becton Dickinson (Franklin Lakes, NJ, USA), diluted in 0.85% saline and introduced into in liquid or into 0.35% Bacto agar with Davis minimal salts [47] supplemented with 0.2mg/ml glucose as the sole and limiting carbon source. The liquid cultures were maintained with 10 ml of the medium in 50 ml flasks. For the 3-d colony experiments, 3ml of bacteria suspended in soft agar were put into the wells of 6-well Costar Macrotiter plates, set in a tray with distilled water to reduce the rate of evaporation.

#### Sampling

The viable cell densities in both the liquid and soft agar cultures were estimated by serial dilution in 0.85% saline and plating on LB agar. The bacteria from the soft agar in each well were taken up with Samco Scientific long transfer pipettes, VWR International, transferred to glass tubes with 10 ml of 0.85% saline. To suspend the bacteria in the soft agar, tubes with the 10 ml of saline and 3 ml of soft agar were disrupted with the long transfer pipettes until the soft agar was fully mixed with saline, and then vortexed for 30 seconds.

### Cell size measurement

To measure the cell sizes in liquid cultures at a certain time point, 5 ml of the experiment culture was centrifuged at 4,000 rpm for 4 min. Excess supernant was then disposed of, so that the cell density of the resuspension of the rest of the culture was at least 10^7^ cells/ml. Cells were then put on a microscope slide (Leica (Wetzlar, Germany) TCS SP8 inverted confocal microscope with live-cell chamber at 60x (HC PL APO 63x/1.40 oil CS2 WD 0.14 mm)), photographed, and their length in the pictures was measured manually using ImageJ (National Institutes of Health, Bethesda, MD). For colony cultures, centrifuge would not separate the agar and the cells. Thus we first dispersed the colony culture and then directly sampled from the mixture. In order to have enough cells per slide to image, the 6-hour old cultures were inoculated with 10^6^ cells/ml. The 14-hour old cultures were inoculated with 1000 cells/ml. The older cultures were all inoculated with 50 cells/ml.

### Numerical solution of the model

The well-mixed Monod model, Eqs (1) and (2), was solve using ode15s MatLab routine. To solve the growth equations Eqs (3)–(5) numerically, we rewrote the equations in spherically symmetric coordinates, and then discretize the space into concentric shells so that the partial differential equations become sets of coupled ordinary differential equations describing dynamics within each shell. These were then solved again using Matlab’s ode15s, with an additional constraint that redistributed the total number of bacteria *N* into a bacterial colony with the constant cell packing density, as in Eq (5), at every time step. That is, each discretized shell of the space had a maximum cellular capacity given by the packing density and the shell volume. The constraint redistributed those cells that overflowed each shell’s capacity to the colony’s edge, but we did not shrink the inner shells when the cells in them started dying. Newly grown cells are first filled in the colony’s current edge shell. If the current edge shell is overflowed, the extra cells are filled in the next shell, and so on.

### Model fitting and confidence intervals estimation

We first fitted the five parameters of the Monod model for the growth in the liquid culture, Eqs (1) and (2). For this we defined the loss function $L=\sum_{i}{\left(N_{i}-N\left(t_{i};g_{\text{max}},m,K,\tau_{\text{lag}},a_{l}\right)\right)}^{2}$, where *N*_*i*_ was the population size (in CFU/ml) in the *i*’th measurement, and *N*(*t*_*i*_; *g*_max_, *m*, *K*, *τ*_lag_, *a*_l_) was the model prediction for the same time and for given parameter values. Note that we did not average measurements at the same *t*, but incorporated all individual observations into the loss function, cf. Fig 4. We optimized L over the five parameters using MatLab’s fmincon. For *K* and *m*, which are small and have large uncertainties, we optimized w. r. t. their logarithms, thus enforcing their positivity (the other parameters were sufficiently constrained by data away from zero even without this reparameterization). The optimization was performed with ten different random initial conditions for the parameters, and the best values from among all the runs were chosen, resulting in the best-fit parameters ${\bar{g}}_{\text{max}},\bar{m},\bar{K},{\bar{\tau }}_{l},{\bar{a}}_{l}$, which we report in Table 1.

To estimate the confidence intervals for these inferences, we bootstrapped the data 1000 times [48]. When re-sampling with replacements for bootstrapping, we resampled separately from the exponential growth region (*t* ≤ 22 hrs) and the saturated region (*t* > 22 hrs), so that the number of data points in each of the regions was fixed in all resampled datasets. We refitted the five growth parameters for each of the resampled data sets. The middle 80% of the best-fit parameter realizations are reported in Table 1 as confidence intervals, and the covariances among the bootstrapped best-fit values are reported in Table 2. Since the sensitivities to the parameters vary widely, and L near its minimum is badly approximated by a quadratic form, we additionally report confidence intervals directly on the model predictions, rather than just the parameters. For this, for each of the 1000 resampled datasets, we calculated the population growth with the best-fit parameters, and the middle 80% of these growth curves are shown as the colored band in Fig 4 (top).

**Table 2:** Covariances and correlations of the fitted parameters.

*g*_max_, hr^−1^	*K*, *μ*g/L	*a*_l_, 10^6^ CFU/*μ*g glucose	lag, hr	*m*, hr^−1^	*a*_c_, hr	*D*, 10^6^ *μ*m^2^/hr	*μ*, CFUs/*μ*m^3^
0.029	0.19	0.0018	0.0041	0.0047	6.0 × 10^4^	0.018	5.1 × 10^4^
*0.99*	3.4	0.022	0.047	0.23	0.11	-0.018	0.0056
*0.022*	*0.015*	0.0049	-0.0021	0.061	0.0023	-0.0012	1.0 × 10^4^
*0.26*	*0.19*	*0.046*	0.041	-0.018	0.0035	-0.021	-2.8 × 10^4^
*0.12*	*0.082*	*0.81*	*0.50*	1.8	0.070	-0.089	9.1 × 10^4^
*0.021*	*0.014*	*0.23*	*0.14*	*0.28*	0.018	-0.012	4.5 × 10^4^
*0.25*	*0.26*	*-0.046*	*-0.33*	*0.041*	*-0.27*	0.12	0.0033
*0.19*	*0.18*	*0.11*	*-0.12*	*0.24*	*0.27*	*0.77*	1.5 × 10^4^

The upper right quadrant shows in Roman font the covariance of the fitted parameters established by bootstrapping (see Materials and methods). The diagonal are the parameter variances. The lower left quadrant shows the correlation coefficients in *Italic*. Units for the parameters are the same as in Table 1. While we report these values, we emphasize that these values must be interpreted with care since posterior distributions of the parameters are sloppy [49] and do not look like multivariate normal distributions. Instead they show long nonlinear ridges of parameters with nearly-equivalent likelihoods.

For fitting the 3-d growth model, Eqs (3)–(5), we write the loss function $L=\sum_{i}{\left(N_{i}-N\left(t_{i};{\bar{g}}_{\text{max}},\bar{m},\bar{K},{\bar{\tau }}_{l},a_{c},D,\mu \right)\right)}^{2}$. This is minimized as above over *a*_c_, *D*, *μ*, with the first four parameters inherited from the optimizations for liquid data. Results of the optimization are shown in Fig 4 (bottom). To establish confidence intervals, we bootstrap the entire analysis pipeline 30 times (the number is limited since parameter optimizations for PDEs describing the nutrient dynamics are computationally costly), resampling both the liquid and the 3-d colony data. While resampling the colony data, we keep the number of data points in each of the three regions constant (exponential, *t* < 24, diffusion-limited, 24 ≤ *t* < 48, and saturated, *t* ≥ 48). Confidence intervals on parameters and model predictions in Fig 4 (bottom) and Fig 5 are then done as explained above. We use the same bootstrapped data sets to estimate the covariances and correlations of the parameters (Table 2). These are evaluated as empirical covariances and correlation coefficients of the best-fit values for the bootstrapped data sets.

## References

[1] Monod J. Recherches sur la croissance des cultures bacteriennes. Hermann; 1942.

[2] Monod J. The growth of bacterial cultures. Ann Rev Microbiol. 1949;3:371–394. 10.1146/annurev.mi.03.100149.002103

[3] Kubitschek HE. Introduction to research with continuous cultures. Prentice-Hall; 1970.

[4] Stewart F, Levin B. Partitioning of Resources and the Outcome of Interspecific Competition: A Model and Some General Considerations. American Naturalist. 1973;107:171–198. 10.1086/282825

[5] Ben-Jacob E, Schochet O, Tenenbaum A, Cohen I, Czirok A, Vicsek T. Generic Modeling of Cooperative Growth-Patterns in Bacterial Colonies. Nature. 1994;368:46–49. 10.1038/368046a0 8107881

[6] Ben-Jacob E, Cohen I, Shochet O, Tenenbaum A, Vicsek T, Czirok A. Cooperative Formation of Chiral Patterns During Growth of Bacterial Colonies. Phys Rev Lett. 1995;75:2899–2902. 10.1103/PhysRevLett.75.2899 10059433

[7] Croze OA, Ferguson GP, Cates ME, Poon WC. Migration of chemotactic bacteria in soft agar: role of gel concentration. Biophys J. 2011;101:525–534. 10.1016/j.bpj.2011.06.023 21806920 PMC3145277

[8] Rudge TJ, Federici F, Steiner PJ, Kan A, Haseloff J. Cell polarity-driven instability generates self-organized, fractal patterning of cell layers. ACS Synth Biol. 2013;2:705–714. 10.1021/sb400030p 23688051

[9] Liu C, Fu X, Liu L, Ren X, Chau C, Li S, et al Sequential establishment of stripe patterns in an expanding cell population. Science. 2011;334(6053):238–241. 10.1126/science.1209042 21998392

[10] Asally M, Kittisopikul M, Rue P, Du Y, Hu Z, Cagatay T, et al Localized cell death focuses mechanical forces during 3D patterning in a biofilm. Proc Natl Acad Sci (USA). 2012;109:18891–18896. 10.1073/pnas.1212429109 23012477 PMC3503208

[11] Wei Y, Wang X, Liu J, Nemenman I, Singh A, Weiss H, et al The population dynamics of bacteria in physically structured habitats and the adaptive virtue of random motility. Proc Natl Acad Sci (USA). 2011;108(10):4047–4052. 10.1073/pnas.1013499108 21325053 PMC3053974

[12] Kim W, Racimo F, Schluter J, Levy S, Foster K. Importance of positioning for microbial evolution. Proc Natl Acad Sci (USA). 2014;111(16):E1639–47. 10.1073/pnas.1323632111 24715732 PMC4000849

[13] Mertz AF, Banerjee S, Che Y, German GK, Xu Y, Hyland C, et al Scaling of Traction Forces with the Size of Cohesive Cell Colonies. Phys Rev Lett. 2012;108:198101 10.1103/PhysRevLett.108.198101 23003091 PMC4098718

[14] Farrell F, Hallatschek O, Marenduzzo D, Waclaw B. Mechanically Driven Growth of Quasi-Two-Dimensional Microbial Colonies. Phys Rev Lett. 2013;111:168101 10.1103/PhysRevLett.111.168101 24182305

[15] Ghosh P, Mondal J, Ben-Jacob E, Levine H. Mechanically-driven phase separation in a growing bacterial colony. Proc Natl Acad Sci U S A. 2015;112(17):E2166–73. 10.1073/pnas.1504948112 25870260 PMC4418877

[16] Witten T, Sander L. Diffusion-Limited Aggregation, a Kinetic Critical Phenomenon. Phys Rev Lett. 1981;47:1400–1403. 10.1103/PhysRevLett.47.1400

[17] Family F. Fractal growth of bacterial colonies. Fractals. 1995;3:869–877. 10.1142/S0218348X9500076X

[18] Lavrentovich MO, Koschwanez JH, Nelson DR. Nutrient shielding in clusters of cells. Phys Rev E. 2013;87 10.1103/PhysRevE.87.062703 PMC4122756 23848711

[19] Kreft JU, Booth G, Wimpenny JW. BacSim, a simulator for individual-based modelling of bacterial colony growth. Microbiology. 1998;144(12):3275–3287. 10.1099/00221287-144-12-3275 9884219

[20] Ward J, King J, Koerber AJ, Croft J, Sockett E. Early development and quorum sensing in bacterial biofilms. J Math Biol. 2003;47:23–55. 10.1007/s00285-002-0190-6 12827447

[21] Eberl H, Parker D, Van Loosdrecht M. A New Deterministic Spatio-Temporal Continuum Model for Biofilm Development. J Theor Med. 2001;3:161–175. 10.1080/10273660108833072

[22] Eberl H, Picioreanu C, Heijnen J, van Loosdrechtb M. A three-dimensional numerical study on the correlation of spatial structure, hydrodynamic conditions, and mass transfer and conversion in biofilms. Chem Eng Sci. 2000;55:6209–6222. 10.1016/S0009-2509(00)00169-X

[23] Duddu R, Bordas S, Chopp D, Moran B. A combined extended finite element and level set method for biofilm growth. Int J Numer Methods Eng. 2008;74(5):848–870. 10.1002/nme.2200

[24] Alpkvista E, Klapper I. A multidimensional multispecies continuum model for heterogeneous biofilm development. Bull Math Biol. 2007;69(2):765–789. 10.1007/s11538-006-9168-7 17211734

[25] Zhang T, Cogan N, Wang Q. Phase Field Models for Biofilms. I. Theory and One-Dimensional Simulations. SIAM J Appl Math. 2008;69:641–669. 10.1137/070691966

[26] Zhang T, Cogan N, Wang Q. Phase-Field Models for Biofilms II. 2-D Numerical Simulations of Biofilm-Flow Interaction. Comm Comput Phys. 2008;p. 72–101.

[27] Phaiboun A, Zhang Y, Park B, Kim M. Survival Kinetics of Starving Bacteria Is Biphasic and Density-Dependent. PLoS Comput Biol. 2015 4;11(4):e1004198 10.1371/journal.pcbi.1004198 25838110 PMC4383377

[28] Picioreanu C, van Loosdrecht M, Heijnen J. Mathematical Modeling of Biofilm Structure with a Hybrid Differential-Discrete Cellular Automaton Approach. Biotechn Bioeng. 1998;58:101 10.1002/(SICI)1097-0290(19980405)58:1%3C101::AID-BIT11%3E3.0.CO;2-M 10099266

[29] Noguera D, Pizarro G, Stahl D, Rittmann B. Simulation of multispecies biofilm development in three dimensions. Water Sci Techn. 1999;39:123 10.1016/S0273-1223(99)00159-6

[30] Saint-Ruf C, Garfa-Traoré M, Collin V, Cordier C, Franceschi C, Matic I. Massive diversification in aging colonies of Escherichia coli. Journal of bacteriology. 2014;196(17):3059–3073. 10.1128/JB.01421-13 24982303 PMC4135658

[31] Redner S. A Guide to First-Passage Processes. Cambridge UP; 2007.

[32] Pierucci O. Dimensions of Escherichia coli at various growth rates: model for envelope growth. J Bacteriol. 1978;135(2):559–74. 355233 10.1128/jb.135.2.559-574.1978 PMC222416

[33] Taheri-Araghi S, Bradde S, Sauls JTT, Hill NSS, Levin PAA, Paulsson J, et al Cell-Size Control and Homeostasis in Bacteria. Curr Biol. 2014;25(3):385–391. 10.1016/j.cub.2014.12.009 25544609 PMC4323405

[34] Simonsen L. Dynamics of plasmid transfer on surfaces. J Gen Microbiol. 1990;136:1001–1007. 10.1099/00221287-136-6-1001 2200839

[35] Ecker RE, Schaechter M. Bacterial growth under conditions of limited nutrition. Annals NY Acad Sci. 1963;102:549–563. 10.1111/j.1749-6632.1963.tb13660.x

[36] Pelletier J, Halvorsen K, Ha BY, Paparcone R, Sandler SJ, Woldringh CL, et al Physical manipulation of the Escherichia coli chromosome reveals its soft nature. Proc Natl Acad Sci. 2012;109(40):E2649–E2656. 10.1073/pnas.1208689109 22984156 PMC3479577

[37] Füchslin HP, Schneider C, Egli T. In glucose-limited continuous culture the minimum substrate concentration for growth, Smin, is crucial in the competition between the enterobacterium Escherichia coli and Chelatobacter heintzii, an environmentally abundant bacterium. ISME J. 2012;6(4):777–89. 10.1038/ismej.2011.143 22030672 PMC3309354

[38] Levin BR. Coexistence of Two Asexual Strains on a Single Resource. Science. 1972;175(4027):1272–1274. 10.1126/science.175.4027.1272 4551427

[39] Chao L, Levin BR. Structured habitats and the evolution of anticompetitor toxins in bacteria. Proc Natl Acad Sci U S A. 1981;78(10):6324–6328. 10.1073/pnas.78.10.6324 7031647 PMC349031

[40] Buchanan R, Klawitter L. The effect of incubation temperature, initial pH, and sodium chloride on the growth kinetics of Escherichia coli O157: H7. Food Microbiology. 1992;9(3):185–196. 10.1016/0740-0020(92)80046-7

[41] van Stroe-Biezen SAM, Everaerts FM, Janssen LJJ, Tacken RA. Diffusion coefficients of oxygen, hydrogen peroxide and glucose in a hydrogel. Analytica chimica acta. 1993;273:553–560. 10.1016/0003-2670(93)80202-V

[42] Koch A. Distribution of cell size in growing cultures of bacteria and the applicability of the Collins-Richmond principle. Microbiology. 1966;45(3):409–417.

[43] Kubitschek H. Growth during the bacterial cell cycle: analysis of cell size distribution. Biophys J. 1969;9(6):792. 4978435 10.1016/S0006-3495(69)86418-0 PMC1367476

[44] Asally M, Kittisopikul M, Rue P, Du Y, Hu Z, Cagatay T, et al Localized cell death focuses mechanical forces during 3D patterning in a biofilm. Proc Natl Acad Sci (USA). 2012;109:18891–18896. 10.1073/pnas.1212429109 23012477 PMC3503208

[45] Wentland EJ, Stewart PS, Huang CT, McFeters Ga. Spatial variations in growth rate within Klebsiella pneumoniae colonies and biofilm. Biotechnol Prog. 1996;12(3):316–321. 10.1021/bp9600243 8652119

[46] Lenski RE, Rose MR, Simpson SC, Tadler SC. Long-term experimental evolution in Escherichia coli. I. Adaptation and divergence during 2,000 generations. American naturalist. 1991;p. 1315–1341. 10.1086/285289

[47] Carlton B, Brown B. Gene mutation in Manual of Methods for General Bacteriology. Gerhardt et al, ed, American Society for Microbiology, Washington, DC 1981;.

[48] Efron B, Tibshirani R. An introduction to the bootstrap. Boca Raton, FL: Chapman & Hall/CRC; 1994.

[49] Gutenkunst RN, Waterfall JJ, Casey FP, Brown KS, Myers CR, Sethna JP. Universally sloppy parameter sensitivities in systems biology models. PLoS Comp Biol. 2007;3(10):1871–1878. 10.1371/journal.pcbi.0030189 PMC2000971 17922568

